# Phase 1 study of concomitant tumor treating fields and temozolomide chemoradiation for newly diagnosed glioblastoma

**DOI:** 10.1093/noajnl/vdae129

**Published:** 2024-07-29

**Authors:** Samuel A Goldlust, Samuel Singer, Lori A Cappello, Ahmad K AlMekkawi, Kangmin D Lee, Anthony C Ingenito, Brett E Lewis, Themba Nyirenda, Hooman Azmi, George J Kaptain

**Affiliations:** Department of Oncology, Hackensack University Medical Center, Hackensack, New Jersey, USA; Department of Oncology, Hackensack University Medical Center, Hackensack, New Jersey, USA; Department of Oncology, Hackensack University Medical Center, Hackensack, New Jersey, USA; Department of Neurosurgery, Saint Luke’s Hospital of Kansas City, Kansas City, Missouri, USA; Department of Neurosurgery, Hackensack University Medical Center, Hackensack, New Jersey, USA; Department of Radiation Oncology, Hackensack University Medical Center, Hackensack, New Jersey, USA; Department of Radiation Oncology, Hackensack University Medical Center, Hackensack, New Jersey, USA; Office of Research Administration, Hackensack Meridian Health, Edison, New Jersey, USA; Department of Neurosurgery, Hackensack University Medical Center, Hackensack, New Jersey, USA; Department of Neurosurgery, Hackensack University Medical Center, Hackensack, New Jersey, USA

**Keywords:** glioblastoma, phase 1, tumor treating fields

## Abstract

**Background:**

Glioblastoma (GBM) is the most common and aggressive primary brain tumor and has limited effective therapies. Tumor treating fields (TTF; Optune Gio^®^) is an FDA-approved device with data supporting a significant survival benefit and minimal toxicity when added to maintenance chemotherapy. Uptake in clinical practice is not universal and might improve if a shorter duration of treatment is feasible. This phase 1 trial was designed to determine the safety and preliminary efficacy of TTF concomitant to chemoradiation.

**Methods:**

Patients with newly diagnosed, histologically confirmed GBM were eligible. Following surgery, patients were treated with TTF concomitant to standard chemoradiation. The device continued through 2 monthly cycles of maintenance temozolomide with imaging and clinical assessments at regular intervals to assess toxicity and response. The primary endpoint was the safety and tolerability of combined modality treatment based upon the incidence and severity of adverse events. Secondary endpoints were overall survival (OS) and progression-free survival (PFS).

**Results:**

Thirteen patients were enrolled. Dermatologic adverse events were frequent but limited to grade 1/2. There was only 1 serious adverse event possibly related to TTF and no patients were unable to complete the prescribed course of multimodality treatment due to TTF-associated toxicity. Twelve patients were evaluable for median and 6-month progression-free survival which were 8.5 months (mo) and 66.7%, respectively. Median and 12 mo overall survival were 16.0 mo and 83.3%, respectively.

**Conclusions:**

TTF can be safely delivered in conjunction with chemoradiation. The potential for a finite TTF course merits further evaluation.

## Lay Summary

Glioblastoma is an aggressive brain cancer. Optune Gio^®^ is a device worn on the scalp that uses alternating electrical fields to stop cancer cells from dividing. Previous studies have shown that patients with glioblastoma live longer when they use Optune Gio^®^ with maintenance chemotherapy after completing combined chemotherapy and radiation. The authors of this study wanted to see if it would be safe to use Optune Gio^®^ earlier in treatment. To do this they treated 13 patients with newly diagnosed glioblastoma with Optune Gio^®^ at the same time as chemotherapy and radiation therapy. Their results show that none of the patients had to stop treatment because of side effects from Optune Gio^®^. The most common side effects related to treatment were skin changes. Approximately 83% of patients remained alive 12 months after starting the treatment.

**Key Points**

- Tumor treating fields and concomitant chemoradiation were well tolerated in newly diagnosed glioblastoma.
- Preliminary efficacy was comparable with historical controls.

**Importance of the Study**

Glioblastoma (GBM) has limited treatment options. The addition of tumor treating fields (TTF) to maintenance temozolomide offers improved survival with manageable toxicity. The use of TTF is not universal and might improve if a shorter duration of treatment is feasible. This single institution, prospective, phase 1 single-arm trial enrolled 13 patients with newly diagnosed GBM. Multimodality treatment with TTF, radiotherapy (XRT) and temozolomide was well tolerated and efficacy was comparable with historical controls. Dermatologic adverse events were limited to grade 1/2, there was only 1 serious adverse event possibly related to TTF and no patients were unable to complete the prescribed course of multimodality treatment due to TTF-associated toxicity. This data supports a phase 2 dose expansion, potentially incorporating contemporaneous data supporting XRT delivery through intact scalp arrays.

Glioblastoma (GBM) is the most common and aggressive primary brain tumor. Long-term survivors are rare, with only 6.9% alive at 5 years from diagnosis,^1^ and more efficacious therapies are needed. In addition to the mainstays of resection, temozolomide (TMZ) chemoradiation and bevacizumab, a more recent addition to the GBM treatment landscape is Tumor Treating Fields (TTF; Optune Gio^®^). This device creates a low-intensity, intermediate frequency (200 kHz) alternating electric field transmitted via transducer arrays worn on the scalp, theorized to induce cancer cell death via interruption of mitosis and subsequent apoptosis.^2,3^ Randomized studies have led to FDA approval of the device in both newly diagnosed^4^ and recurrent GBM.^5^ However, despite minimal toxicity and a 4.9 month (mo) survival advantage with the addition of TTF to maintenance TMZ, use of the device in clinical practice is not universal.^4,6,7^ While clinical trials of TTF have been diligent in assessing quality of life (QOL),^8,9^ such subjective assessments are limited by selection bias. Logistical considerations and lack of a finite end point to treatment may dissuade physicians and patients alike.

Distinct from the schema of TTF initiation following completion of chemoradiation which led to FDA approval, preclinical data indicates that TTF concomitant to radiotherapy (XRT) may enhance cytotoxic impact through synergistic inhibition of DNA replication and repair pathways.^10–12^ Accordingly, the addition of TTF earlier in the disease course may offer improved efficacy and the potential to derive a survival benefit from a shorter treatment duration. The present prospective study was designed to assess the safety and preliminary efficacy of combined XRT, TMZ and TTF at diagnosis.

Recent data from single-arm pilot studies at other institutions demonstrated the safety of similar combined modality approaches used in the present study,^13,14^ and a phase 3 randomized trial has completed accrual (NCT04471844).

## Methods

### Trial Design

This study was designed as a single institution, prospective, single-arm trial of 10 patients with newly diagnosed GBM. Additional patient (s) could be enrolled to replace those who discontinued treatment on the protocol for reasons unrelated to TTF-associated toxicity.

### Trial Registration

The trial is registered with ClinicalTrials.gov (NCT03232424).

### Participants

Adults (≥18 years) with KPS ≥ 70 and newly diagnosed, histologically confirmed supratentorial GBM were eligible. All participants underwent maximum safe resection as determined by the treating neurosurgeon. Key inclusion criteria included: life expectancy of at least 3 months, adequate marrow, hepatic, and renal function. Patients were excluded if they received prior GBM treatment aside from resection, if unhealed scalp wounds or implanted devices were a contraindication to TTF, or if comorbid medical or psychiatric illness was deemed by the treating investigator to place the patient at increased risk. The protocol and informed consent forms were approved by the institutional review board. Written informed consent was obtained from all patients.

### Treatment and Evaluations

**Figure 1.**
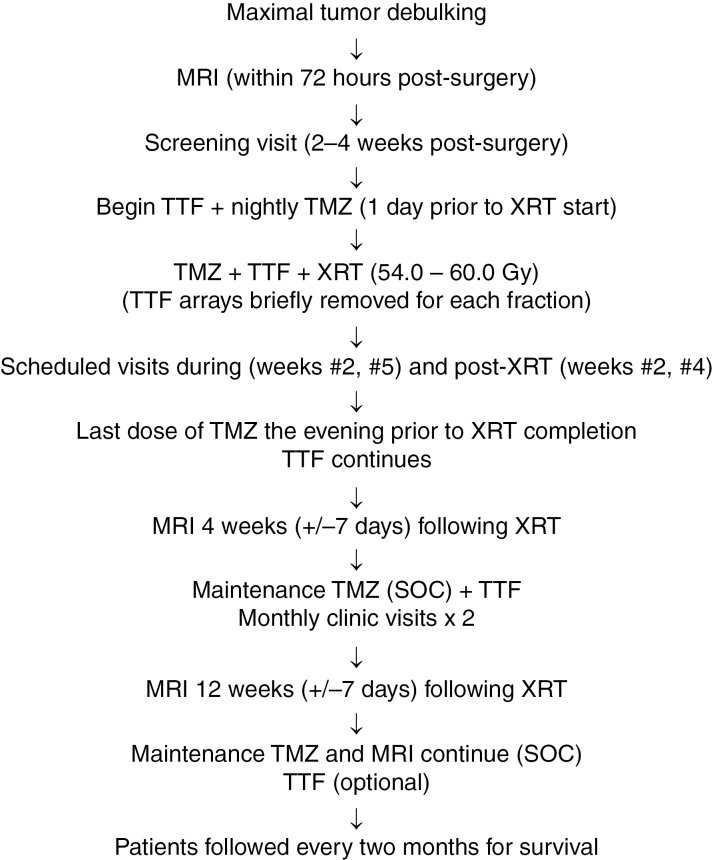
Trial schema. Abbreviations: SOC: standard of care; TMZ: temozolomide; TTF: tumor treating fields; XRT: radiotherapy.

The trial schema is detailed in Figure 1. Patients underwent a gadolinium-enhanced brain MRI within 72 h of surgery and a screening visit 2–4 weeks postoperatively. The extent of resection was recorded as biopsy, sub-total, or gross-total resection based upon residual contrast-enhancing tumor. O^6^-methylguanine-DNA methyltransferase (MGMT) promoter methylation status and isocitrate dehydrogenase (IDH) mutational status were assessed provided there was sufficient tissue.

Using the postoperative MRI and proprietary planning software furnished by the study sponsor (NovoTAL^™^) the investigator assessed and inputted properties of the patient’s head and tumor anatomy to design a transducer array map to be used during treatment. Each map was reviewed by a second investigator prior to the start of treatment.

The day prior to the XRT start, patients underwent device education and initiated TTF. Radiotherapy commenced 5 weeks after the definitive surgical procedure (±1 week), to a total dose of 54.0–60.0 Gy, delivered in 1.8–2.0 Gy fractions over 6–7 weeks. Target volumes were determined utilizing all available imaging studies that best delineated the extent of the disease. Fusion image registration for treatment planning was utilized as possible with either 3D conformal or intensity-modulated radiation therapy.

Temozolomide 75 mg/m^2^ was dosed nightly during XRT to minimize patient inconvenience and to mitigate potential toxicity. Tumor-treating fields were worn continuously, removed during XRT and replaced under staff supervision immediately following each fraction. Patients were encouraged to wear the device continuously, but no less than 18 h per day, and TTF usage time was assessed monthly. During XRT and for 12 weeks to follow, patients had study visits at regular intervals for a physical examination, serum laboratories and to assess toxicity and device compliance. Management of dermatologic scalp toxicity was coordinated with planned array changes and followed guidelines as outlined by Lacoutre ME and colleagues.^15^

Brain MRI was obtained at 4 weeks (±7 days) and 12 weeks (±7 days) following completion of XRT, and maintenance TMZ recommenced in 5/28 day cycles as per standard of care. Temozolomide and TTF continued until the final study visit, or until disease progression or unacceptable toxicity. Thereafter, TMZ, MRI and response assessments continued as per the standard of care. Following the final study visit, the patient was followed at a minimum of every 2 months for survival. If the patient was free of unacceptable toxicity attributable to TTF at study completion (week 12 post-XRT), they were offered the opportunity to continue the device, but without obligation to do so.

The primary study endpoint was the safety and tolerability of combined modality treatment with XRT, TMZ, and TTF, based upon the incidence and severity of adverse events. Secondary endpoints were overall survival (OS) and progression-free survival (PFS) from initiation of TTF. Response was assessed as per the Response Assessment in Neuro-Oncology (RANO) criteria^16^ and confirmed by a second investigator.

Adverse events (AE) were assessed at each visit and defined by the Common Terminology Criteria for Adverse Events version 4 (CTCAEv.4), with the exception of dermatologic scalp toxicity, which was assessed using criteria as per Lacouture and colleagues.^15^ Severity and causality of AEs were determined and classified by the treating investigator as unrelated, unlikely, possibly, probably or related to study treatment. The AE reporting period began at the initiation of treatment with TTF and was collected for 2 months following treatment termination. All AE were followed until resolution or until the investigator assessed the AE as chronic.

Any serious adverse event (SAE) deemed probably related or related was cause for immediate cessation of the attributable modality. Visits, assessments, and nonattributable treatments were continued after such an event. Unacceptable toxicity included the occurrence of device related SAE or clinical and functional deterioration considered by the investigator to be prohibitive of continuing combined modality treatment.

The trial was to be discontinued if 3 or more patients suffered an SAE deemed probably or definitely related to TTF or if 3 or more patients were unable to complete the intended course of combined modality treatment for toxicity deemed probably related or related to TTF.

### Statistics

Safety analyses were descriptive in nature. Overall survival was defined as the time from first treatment on protocol until death. Progression-free survival was defined as the time from first treatment on protocol until progression of disease or death. If a final event was not recorded, dates were censored as of the last radiographic analysis and/or confirmation of survival. Survival results were calculated using Kaplan–Meier methodology.

## Results

Demographics and treatment are summarized in Table 1. Between July 2017 and April 2020, 13 patients (6 women) were enrolled at a single institution (Hackensack University Medical Center). Two patients withdrew consent, one prior to initiation of treatment and another mid-course, and 2 additional patients could not complete treatment on protocol due to a craniectomy defect and cytopenias. Among the 12 patients eligible for assessment of toxicity and efficacy, the median age was 58 (range 21–69) and the median Karnofsky Performance Status (KPS) was 90 (range 70–90). Extent of resection was gross total in 50% (6/12) and subtotal in the remaining 50%. Tumor from 3 patients (3/12; 25%) harbored a methylated MGMT promoter and among those patients with sufficient tissue for analysis, 1 (1/8; 13%) tumor harbored mutant IDH. Tumor Treating Fields compliance was at or above goal (≥75%) in 7 patients (58%).

**Table 1.** Demographics and Treatment

Patient #	1	2	3	4	5	6	7	8	9	10	11	12	13
Age	68	69	47	54	67	60	64	64	56	48	21	36	60
Sex	M	F	M	M	F	M	F	M	F	M	F	F	M
KPS	90	90	90	80	70	90	90	90	80	80	90	80	80
MGMT	UM	M	UM	UM	UM	UM	UM	M	UM	UM	UM	M	UM
IDH	UK	UK	MT	UK	WT	WT	WT	WT	WT	WT	WT	WT	WT
EOR	ST	GT	ST	GT	ST	GT	ST	ST	GT	GT	GT	ST	GT
Weeks on TTF	8	1	171	19	27	44	37	18	73	2	20	44	NA
TTF compliance ≥ 75%	N	Y	Y	N	Y	N	Y	N	Y	N	Y	Y	NA
Scalp toxicity	N	N	Y	Y	Y	Y	Y	Y	Y	N	Y	Y	NA
Monthly cycles of TMZ	5	0	6	6	3	6	0	2	6	1	8	9	0
OR	SD	SD	SD	SD	PD	SD	SD	SD	SD	SD	SD	SD	NA
Completed TTF/XRT/TMZ	Y	N^a^	Y	Y	Y	Y	N^b^	Y	Y	N^c^	Y	Y	N^c^

Abbreviations: EOR: extent of resection; GT: gross total resection; IDH: isocitrate dehydrogenase; KPS: Karnofsky performance status; M: methylated; MGMT: O^6^-methylguanine-DNA-methyltransferase; MT: mutant; OR: overall response; PD: progressive disease; SD: stable disease; ST: subtotal resection; TMZ: temozolomide; TTF: tumor treating fields; UK: unknown; UM: unmethylated; WT: wild type; XRT: radiotherapy.

^a^Could not continue TTF due to craniectomy.

^b^Could not complete TMZ and XRT due to cyopenias.

^c^Withdrew consent.

Treatment emergent and scalp-specific AE are summarized in Tables 2 and 3, respectively. The most common AE were fatigue (8 patients), thrombocytopenia (6 patients), neutropenia (6 patients), and nausea (5 patients), and deemed related to underlying disease, XRT or temozolomide. Nine patients (9/12; 75%) experienced scalp toxicity deemed at least possibly related to TTF. This was low grade, responded to topical therapy, array repositioning or self-limited in all cases, and lead to no treatment delays or discontinuation. There was a total of 11 serious adverse events (SAE), all deemed unrelated to TTF save for 1 episode of fall deemed possibly related to TTF (Table 4).

**Table 2.** Treatment Emergent Adverse Events^a^

	Grade 1	Grade 2	Grade 3	Grade 4
Blood and lymphatic system disorders				
Anemia			1	
Gastrointestinal disorders				
Nausea	2	3		
Constipation	2	1		
Vomiting	3	1		
General disorders				
Fatigue	3	4	1	
Injury, poisoning, and procedural complications				
Fall	1			
Investigations				
Blood bilirubin increased	1			
Weight loss	1			
Decreased lymphocyte count		1	2	
Alanine aminotransferase increased	2			
Aspartate aminotransferase increased	2			
Platelet count decreased	1	2	1	2
Neutrophil count decreased	2	1	2	1
Metabolism and nutrition disorders				
Anorexia	3	1		
Nervous system disorders				
Headache	3	1		
Dysgeusia	1	1		
Psychiatric disorders				
Insomnia	1			
Skin and subcutaneous disorders				
Rash maculo-papular			1	

^a^Deemed at least possibly related to treatment.

**Table 3.** Treatment Emergent Dermatologic Adverse Events^a^

	Grade 1	Grade 2
Dermatitis	4	2
Skin Erosion	3	
Pruritis		1

^a^Deemed at least possibly related to TTF.

**Table 4.** Serious Adverse Events

	Grade	Relationship to TTF
Immune system disorders		
Allergic reaction	3	Unrelated
Infections and infestations		
Lung infection	3	Unrelated
Urinary tract infection (3)	3	Unrelated
Wound infection	3	Unrelated
Injury, poisoning and procedural complications		
Fall	3	Possible
Investigations		
Platelet count decreased	4	Unrelated
Nervous system disorders		
Seizure	1	Unrelated
Seizure	2	Unrelated
Seizure	3	Unrelated

**Figure 2.**
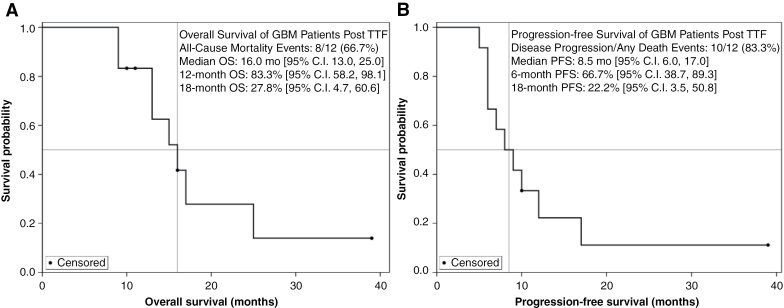
Kaplan–Meier analysis of overall survival (OS) (A) and progression-free survival (PFS) (B).

Among the 6 patients with measurable disease at baseline, there were no responders. The best response was stable disease in 92% (11/12). After 10 progression events, the median PFS was 8.5 mo (95% CI: 6.0, 17.0), and the 6-mo PFS rate was 66.7%. Median OS was 16 mo (95% CI: 13.0, 25.0) and 12 mo OS rate was 83.3% (Figure 2).

## Discussion

This phase 1 trial was designed primarily to determine the safety of combined modality treatment of chemoradiation and TTF for newly diagnosed GBM. Although more frequent than reported in the pivotal trial leading to TTF approval (75% vs. 52%),^4^ dermatologic adverse events were limited to grade 1/2 and manageable with topical intervention, lead repositioning or self-limited. There was only 1 SAE possibly related to TTF and no patients were unable to complete the prescribed course of multimodality treatment due to TTF-associated toxicity. Preliminary efficacy endpoints were comparable to historical controls.

The addition of TTF to maintenance temozolomide offers a 4.9 mo OS benefit and improved survival rate at 5 years (13% vs. 5%).^4^ However, despite this benefit, only 30% of academic neuro-oncologists view the treatment as a definitive part of standard of care, with the most frequent barrier to use as “patient choice for convenience, compliance or other.”^7^ Moreover, a retrospective analysis of patients offered TTF for newly diagnosed GBM demonstrated an acceptance rate of 36%. The most common reason to forgo treatment was cited as “personal reasons (eg head shaving, visibility of the device, and noncompatibility with work).”^6^

TTF are hypothesized to disrupt mitosis through microtubule depolymerization and aberrant spindle formation, precluding the formation of viable daughter cells.^17^ Preclinical models of nonsmall cell lung cancer and GBM have explored TTF + XRT concomitantly or in close temporal succession, and demonstrated synergistically increased apoptosis, DNA damage, mitotic abnormalities, as well as suppression of cell migration and invasiveness.^10–12^

In light of data to support potential synergy of combined modality treatment, and limited available therapies to treat this aggressive tumor, the present study serves as a cornerstone for future studies to determine if benefit might be safely derived from TTF used for a defined interval earlier in the treatment course. While the reasons for forgoing the device described above would not change with the proposed schema, acceptance might evolve with an abbreviated treatment course.

Concurrent to the present study, mixed reports emerged of a theoretical increase in skin toxicity with the addition of TTF to chemoradiation, however, without significant compromise in target volume coverage.^18–21^ As such, a pilot study was completed using “scalp sparing radiation” in conjunction with TTF and temozolomide which demonstrated safety and feasibility.^14^ This technique defines a 5 mm scalp thickness as an avoidance structure during planning and delivers XRT through the TTF scalp arrays, obviating the need for daily array changes. A multicenter phase 3 protocol of the same paradigm recently completed accrual, using TTF and TMZ in the maintenance setting as the comparator arm (NCT04471844).

Limitations of the present study include small sample size, absent central pathology and imaging review, and inclusion of patients with IDH mutant (or unknown) tumors which have since been reclassified as distinct subset following conception of the study,^22^ to be addressed in phase 2. Moreover, 2 patients withdrew consent and 42% of patients did not achieve the recommended compliance threshold of 75%. It remains to be determined in dose expansion whether compliance might improve with data to support XRT delivery through the radiation arrays, thereby avoiding a cumbersome component of the present experimental design.

## Data Availability

Data generated in this study will be made available upon reasonable request by contacting the corresponding author.
